# Does Divergence in Habitat Breadth Associate with Species Differences in Decision Making in *Drosophila Sechellia* and *Drosophila Simulans*?

**DOI:** 10.3390/genes11050528

**Published:** 2020-05-09

**Authors:** Madeline P. Burns, Frederick D. Cavallaro, Julia B. Saltz

**Affiliations:** Department of Biosciences, Rice University, Houston, TX 77005, USA; fdc2@rice.edu (F.D.C.); julia.b.saltz@rice.edu (J.B.S.)

**Keywords:** decision making, habitat breadth, *Drosophila*, genotypic variation

## Abstract

Decision making is involved in many behaviors contributing to fitness, such as habitat choice, mate selection, and foraging. Because of this, high decision-making accuracy (i.e., selecting the option most beneficial for fitness) should be under strong selection. However, decision making is energetically costly, often involving substantial time and energy to survey the environment to obtain high-quality information. Thus, for high decision making accuracy to evolve, its benefits should outweigh its costs. Inconsistency in the net benefits of decision making across environments is hypothesized to be an important means for maintaining variation in this trait. However, very little is known about how environmental factors influence the evolution of decision making to produce variation among individuals, genotypes, and species. Here, we compared two recently diverged species of *Drosophila* differing substantially in habitat breadth and degree of environmental predictability and variability: *Drosophila* *sechellia* and *Drosophila* *simulans*. We found that the species evolving under higher environmental unpredictability and variability showed higher decision-making accuracy, but not higher environmental sampling.

## 1. Introduction

Decision making is an important behavior involving the evaluation of cues in the environment in order to select an option among one or more alternatives [1,2,3]. Decision making is involved in many behaviors contributing to fitness, such as habitat choice, mate selection, and foraging [4]. Because of this, it follows that maximizing the fitness outcomes of decision-making events should be under strong selection, i.e., that individuals should make “accurate” decisions. However, what is actually observed in nature is a great deal of variation in decision-making outcomes [3,5,6,7,8]. Animals frequently make decisions failing to result in the most beneficial fitness outcomes (i.e., seemingly “inaccurate” decisions), even under circumstances where the optimal decision appears obvious to human observers, or when making the wrong decision can have severe fitness consequences [9,10,11]. So why do we observe this variation in decision making, and how is natural selection acting on this trait to influence the evolution of decision making?

Previous work has demonstrated that highly accurate decision making is costly, both in terms of time and energy [12,13,14,15]. A decision is considered “highly accurate” if the option most beneficial for fitness is selected above all available alternatives. Maximizing the accuracy of a particular decision often involves surveying the environment to obtain information, a process which takes time and energy, and can expose animals to various risks (such as the risk of predation) [3]. Any time and energy spent on information gathering and processing is no longer available for maximizing other aspects of fitness. Evidence for this cost of decision making has been documented in a speed/accuracy tradeoff observed in the flower foraging strategies of bumblebees [3], where “hasty” individuals maximize foraging efficiency through faster decisions but show lower accuracy in selecting flowers lacking their predator, crab spiders. “Cautious” individuals exhibit higher accuracy in predator avoidance, but have lower foraging efficiency, due to the increased sampling time required to obtain information [3]. Additional evidence for the cost of decision making has been observed in a speed/accuracy tradeoff in decision making in great tits [7], as well as a tradeoff between developmental stress and female song choice in songbirds, where females raised in small broods with less developmental stress showed significantly stronger preference for male songs compared to females raised with more developmental stress [8]. 

Because maximizing the fitness outcome of a decision-making event is costly, highly accurate decision making is hypothesized to be favored in unpredictable or risky environments (where there is more variance in potential fitness outcomes), or in environments where the fitness cost of making a wrong decision is particularly high [3,5]. However, very little is currently known about how differences in environmental predictability and variability, often a by-product of species’ evolutionary histories, influence the evolution of decision making [3,12,16,17]. In unpredictable or risky environments, or in high-fitness-cost environments, increased information gathering and decision-making accuracy should be favored, as the benefits of collecting and processing information are expected to outweigh the costs [18]. Environmental predictability is lower in environments that are inherently more complex (have a greater breadth of cues or options) and variable (cues or options vary across time or space) [18,19]. In predictable or low-risk environments, less time and energy should be spent on information gathering, and “rules of thumb” or “good enough” approaches to decision making should be favored [20]. Previous work has shown some evidence for this prediction, in that guppies from high-predation environments exhibited slower decision making and higher accuracy, relative to their counterparts from low-predation environments [5]. However, most examples of this trend have been observed between populations of a given species. There is currently a lack of understanding of how environmental predictability/risk and potential fitness costs contribute to variation in decision making both within and between species, or how species diverge in decision making when they evolve in environments differing in predictability or risk.

To investigate whether divergence in environmental predictability and risk is associated with differences in decision making, we compared two closely related species—one generalist and one specialist—that differ considerably in habitat breadth, and thus the degree of environmental predictability and variability experienced in nature. We predict that generalist species experiencing greater environmental unpredictability and variability should exhibit higher environmental sampling through increased exploratory behavior, and higher decision-making accuracy, relative to their specialist counterparts. Additionally, we predict that the generalist species should have more genotypic variation in exploratory behavior and decision-making accuracy than the specialist species. To address our hypotheses, we compared two closely related species of *Drosophila* recently diverged in habitat use: *Drosophila sechellia* and *Drosophila simulans*, investigating species, sex, and genotypic differences in exploratory behavior (one mechanism of environmental sampling) and habitat choice (an example of decision making).

## 2. Materials and Methods 

### 2.1. *Drosophila sechellia* and *Drosophila simulans*: Closely Related Fruit Fly Species that Differ Substantially in Habitat Breadth

*D. sechellia* and *D. simulans* are recently diverged, with previous work estimating divergence times ranging from 250,000 to 413,000 years ago [21,22,23]. Being so closely related, these sister species can produce viable/sterile male F1 offspring and viable/fertile female F1 offspring [24,25,26]. 

While closely related, these species differ substantially in the degree of environmental variability experienced in nature. *Drosophila* habitat and food are synonymous, in that the ephemeral rotting fruit patches these animals live on serve as both their source of food as well as where they mate and lay eggs, and spend most of their time [27]. As a habitat generalist, *D. simulans* has a broad potential range of habitat options across time and space (depending on the region or time of year), and experiences greater environmental variability relative to *D. sechellia*, a habitat specialist that has evolved to feed and breed preferentially on the *Morinda citrifolia* (noni) fruit [23,24,26]. While *D. sechellia* has specialized on the noni fruit, which is ubiquitous in the Seychelles and present year-round, noni is toxic to other species of *Drosophila* (including *D. simulans*) [24]. Therefore, these species have evolved distinct habitat preferences; the “accurate” habitat choice in regard to fitness outcome differs for each species. For the specialist, *D. sechellia*, noni is the “accurate” choice, while for the generalist, *D. simulans*, “plain” fruit (i.e., not noni) is the “accurate” choice. Additionally, because *D. sechellia* is an island specialist diverged from *D. simulans*, genotypic variation is predicted to be higher in *D. simulans* than in *D. sechellia*.

### 2.2. Genotypes

In addition to species comparisons, we also investigated genotypic variation and sex differences in exploration and decision making within species. Isofemale lines (hereafter “genotypes”) of *D. sechellia* and *D. simulans* were graciously provided by D. Matute in 2016. The *D. sechellia* genotypes (13 total) were collected from various locations across the Seychelles, while the *D. simulans* genotypes (11 total) were collected from varied locations across central and southern Africa (Nairobi, Namibia, and Zambia) and Madagascar ([23,28], pers.comm.). Each genotype was established using a single wild female and inbred thereafter; therefore, individuals of the same genotype are more genetically similar to one another than to other genotypes. Thus, these genotypes collected from a range of wild populations represent a broad range of natural genetic diversity.

### 2.3. Rearing

To rear flies for experiments, 10 virgin females were mated to 10 males of the same species and genotype and placed in vials containing standard fly medium (composed of cornmeal, corn syrup, malt sugar, dead yeast, soy flour, tegosept (methyl paraben), proprionic acid, and phosphoric acid). Newly-eclosed virgin male and female F1 offspring were collected under light CO2 anesthesia on day 15, and then housed individually in vials containing standard fly medium. These experimental flies were allowed to recover from anesthesia for three days prior to beginning the exploratory behavior and habitat choice assays. Flies were not deprived of food prior to trials due to concerns that starvation could influence energy levels, exploratory behavior, or habitat choice, as some previous work has shown that flies with less available search time prior to starvation are more likely to settle for less preferred habitats [29]. 

### 2.4. Measuring Environmental Sampling (Exploratory Behavior)

**Figure 1:**
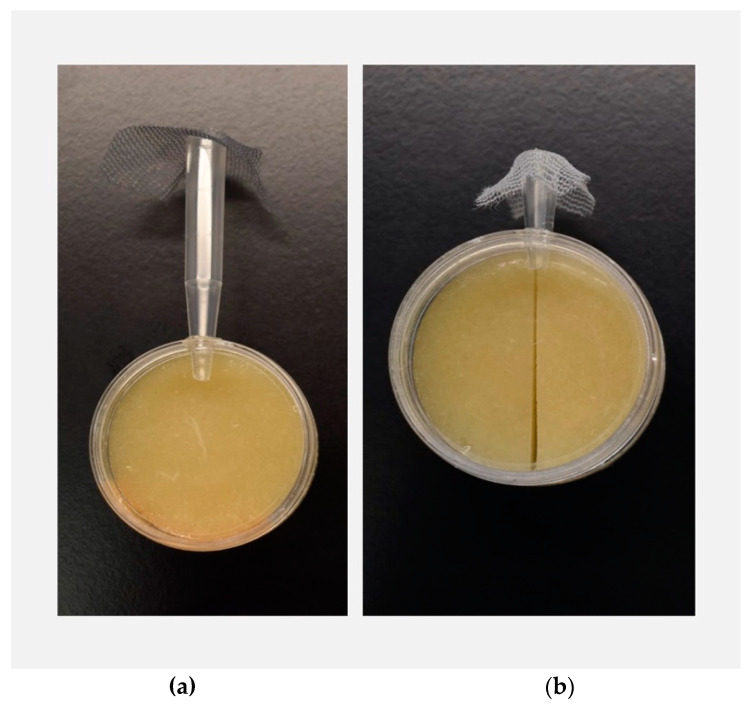
Environmental sampling (exploratory behavior) assay (**a**) and decision-making (habitat choice) assay (**b**): single flies were aspirated into the pipette tips, which were fitted into a small hole in the arena with plain fly food. A mesh barrier prevented each fly from escaping the pipette tip in the other direction. For the environmental sampling assay, each fly had the option to emerge into the arena or remain in the pipette tip. Each fly’s latency to emerge into the arena (in seconds) was recorded. For the decision-making assay, flies were allowed to emerge into the arena on their own accord and were then scan sampled every ten minutes over two hours, and food substrate choice of each fly was recorded.

To compare variation in environmental sampling between *D. sechellia* and *D. simulans*, we investigated differences in exploratory behavior, i.e., the amount of time it took for flies to emerge from a shelter into a novel environment. Willingness to emerge from a shelter into a novel environment has previously been considered a reliable measure of exploratory behavior [30,31,32,33], with greater time to enter the new environment interpreted as low exploratory behavior. Individual flies were gently aspirated into a 1 mL pipette tip (the shelter) and allowed to emerge on their own accord into a petri dish arena (the novel environment). The arenas consisted of one petri dish containing plain fly food medium (consisting of a standard recipe of agar, malt sugar, inactive dry yeast, and deionized water) covered by a second petri dish (acting as a lid), sealed together with tape to create a self-contained arena (Figure 1a). To prevent escape of the fly during the observation period, the large end of the pipette tip was covered in mesh. During the trial, each fly had the option of either remaining in the pipette tip shelter (which lacked food) or emerging from the shelter into the novel arena (with food). Emergence time was measured in seconds over a 5 h time period. At the conclusion of the 5 h observation period, any flies that had not yet emerged were gently moved from the pipette tips into the arenas, and their recorded emergence time was capped at 18,000 s.

### 2.5. Measuring Decision Making (Habitat Choice)

To compare decision-making accuracy between *D. sechellia* and *D. simulans*, the same individual flies evaluated for exploratory behavior were gently aspirated into a shortened pipette tip and allowed to emerge on their own accord into a small petri dish arena (Figure 1b) The arena contained two ecologically relevant food substrate options: plain fly food medium (consisting of a standard recipe of agar, malt sugar, inactive dry yeast, and deionized water), and imitation *M. citrifolia* (noni) fruit medium. Imitation noni food medium was created by adding both octanoic and hexanoic acids to the plain fly food medium, based on a recipe developed by I. Dworkin and C. Jones [34]. Previous work has extensively demonstrated that both octanoic and hexanoic acids are responsible for the toxicity of noni and are involved both in attracting *D. sechellia* and repelling *D. simulans*, thereby providing a good proxy for noni [24,35,36,37,38,39]. Plain fly food medium and imitation noni medium were used in lieu of real fruit patches to ensure that every fly was faced with an identical choice. Previous work has demonstrated that the levels of octanoic and hexanoic acids in noni can vary substantially depending on fruit ripeness, so use of imitation food substrates allowed us to carefully reproduce foods with a molecularly defined composition [24,35]. 

Shortened pipette tips were used for introducing the flies into the habitat choice arenas in this stage of the experiment because they significantly reduce the emergence time of the flies, relative to the 1 mL pipette tips used during the exploratory behavior stage. Habitat choice arenas consisted of one petri dish containing the two food substrate halves, which were cut to be flush and covered by a second petri dish (acting as a lid), sealed together with tape (Figure 1b). The relative location of each of the food substrate halves was varied, such that there was a 1:1 ratio of habitat choice arenas with imitation noni on the left-hand side and plain on the right-hand side, and imitation noni on the right-hand side and plain on the left-hand side. Individuals were assigned randomly to habitat choice arenas. Following emergence into the arena, immediate food choice was recorded. Flies were then scan sampled, with food choice recorded for each fly every 10 min over the course of a 2 h observation period. In total, we collected 13 habitat choice observations for each individual fly. Some previous work has demonstrated that animals often exhibit higher discrimination accuracy in free choice tests (which offer the option to opt out of making a choice) than in forced choice tests (which lack the option to opt out, and thus force a choice) [40,41,42,43]. However, previous work has also shown that the importance of free choice in discrimination accuracy increases with task difficulty [41]. Because of the simplicity of our habitat choice assay and the substantial ecological relevance of our stimuli, we opted for a forced choice test.

### 2.6. Replication

A total sample size of 526 individuals were measured for both the exploratory behavior and habitat choice experiments. For each of the 24 genotypes, a range of 5 to 22 males and a range of 5 to 16 females were measured. The number of replicates varied between genotypes because of variation in the availability of flies on the day of testing. For exploratory behavior via emergence time, each fly (*N* = 526) was observed for a 5 h time block, totaling in 2630 h of observation. For decision making via habitat choice, each fly (*N* = 526) was observed for a 2 h time block, totaling 1052 hours of observation.

### 2.7. Analysis

All analyses were conducted in R version 3.6.1 (Vienna, Australia) (R core team 2019).

#### Exploratory Behavior

For exploratory behavior, our goal was to investigate species, sex, and genotype differences in willingness to emerge from a shelter (pipette tip) into a novel environment (petri dish arena containing food). To do so, we used a mixed-effect proportional hazards model in the coxme package [44]. Cox proportional hazards models are often used to investigate how various fixed and random predictor variables modify an underlying hazard function [45] and are particularly useful for censored data. Because our emergence trials were capped at 5 h, any individuals that had not yet emerged by the end of the trial were assigned an emergence time of 18,000 sec, meaning the data are right censored. Cox proportional hazards models allowed us to test how species and sex affected the “hazard” of leaving the shelter to emerge into the novel environment, while also taking into consideration the right-censoring of our data set. 

Species and sex were included as fixed predictor variables and genotype and trial date were included as random predictor variables. Censoring was modeled using the survival package in R [44] to include a “status” variable indicating whether each measurement was right censored or not. Initial models showed no evidence of non-proportional hazards (global *p*-value = 1.00), indicating that our data set met the assumptions of cox proportional hazards models. Cox proportional hazards models were run for both a main effects-only model (Species + Sex) and a two-way fixed-effects interaction model (Species*Sex). The AIC model comparison indicated that the best summary model was the one with main effects only (Species + Sex), with a delta AIC of 2.0 between the first- and second-best models. This final model (Species + Sex) was used for estimating the effect of genotype. To calculate *p*-values for the fixed effects, we used Type III Wald Chi-square tests implemented in the car package [46]. To test the significance of genotype, we used a likelihood ratio test. To further investigate genotypic variation within species, we ran additional follow-up models separately for each species (with sex included as the fixed predictor variable and genotype and trial date included as the random predictor variables) and tested the significance of genotype using a likelihood ratio test.

### 2.8. Decision Making

Our goal was to investigate whether species, sexes, and/or genotypes differed in habitat choice when presented with the choice between two ecologically relevant food substrates: plain fruit medium or imitation noni medium.

### 2.9. Measuring and Calculating Habitat Choice 

Habitat choice measurements were calculated by averaging the proportion of time each fly spent on the imitation noni medium (with a plain fruit medium observation arbitrarily given a value of 0, while an observation on imitation noni was given a value of 1). For observations where the fly was located in the middle of the two options and failed to make a clear decision between the mediums, no decision was indicated, and the observation was not included in the final habitat choice calculation. This situation was rare—out of 6659 total observations, 261 observations were removed because of failure to choose, resulting in a final total of 6398 observations used for the analysis. 

Thus, to generate a habitat choice measurement for each individual, the number of observations on noni were totaled and then divided by the total number of 13 observations (minus any failures to choose between the two mediums). Habitat choice values at or closer to 0 indicated higher preference for plain medium, while values at or closer to 1 indicated higher preference for the noni medium. Habitat preference values of 0.5 indicated no observable preference for either the plain or noni substrates. 

### 2.10. Decision Making: Species and Sex Differences in Habitat Choice

To investigate whether species differed significantly in habitat choice, we ran a generalized linear mixed model in the lme4 package in R [47], testing the effect of species on habitat choice. Generalized linear mixed models provide a flexible approach to analyzing non-normal data when random effects are present [47]. 

Species, sex, and arena food orientation (whether imitation noni was on the right or left-hand side) were included as fixed effects. Random effects were included to account for the non-independence of genotype as well as the non-independence of the 13 habitat choice observations taken for each individual fly. A random effect was also included for the trial date to indicate which flies were tested on the same day. Model slopes were fitted to a binomial distribution. Generalized linear mixed models were run for all two-way fixed-effects interactions (Species*Sex; Species*Arena Orientation; Sex*Arena Orientation), a three-way fixed-effects interaction (Species*Sex*Arena Orientation), and no interactions (Species + Sex + Arena Orientation). Using AIC model comparison, the best summary model included (Species*Sex + Sex*Arena Orientation), with a delta AIC of 2.2 between the first- and second-best models. The best summary model (Species*Sex + Sex*Arena Orientation) was also used for estimating the effect of genotype. To calculate p-values for the fixed effects, we used Type III Wald Chi-square tests implemented in the car package [46]. In the final model, we tested the significance of genotype using a likelihood ratio test. To further investigate genotypic variation within species, we ran additional follow-up models separately for each species (with sex and arena orientation included as the fixed predictor variables, and genotype and trial date included as the random predictor variables) and tested the significance of genotype using a likelihood ratio test.

## 3. Results

### 3.1. Sexes, but not Species, Differ in Exploratory Behavior

**Figure 2:**
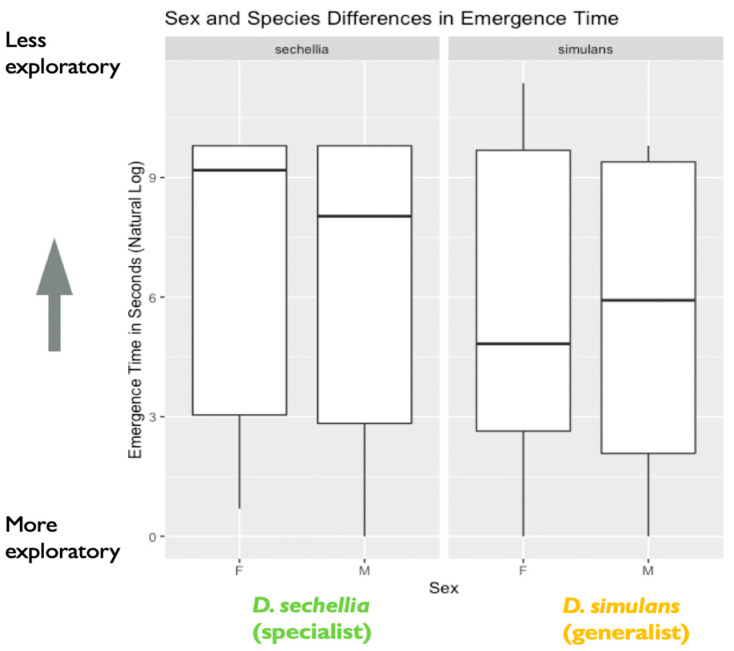
Sex and species differences in emergence time (in seconds): the plot represents species and sex differences in emergence time (in seconds), as noted in the main text. Data are from the first experiment measuring exploratory behavior as a mechanism for environmental sampling. The y-axis represents the natural log of emergence time in seconds. A lower emergence time indicates higher exploratory behavior, while a higher emergence time represents lower exploratory behavior (*N* = 526 flies). The box in the plot represents the inter-quartile range of scores, including the middle 50% of scores for the group, with the median score represented by the middle quartile mark (or line). The whiskers represent the score ranges outside the inter-quartile range, including the top 25% of scores for the group in the upper whisker, and the bottom 25% of scores in the lower whisker. Our findings indicated significant sex, but not species, differences in exploratory behavior, with males showing significantly shorter emergence time than females.

**Figure 3:**
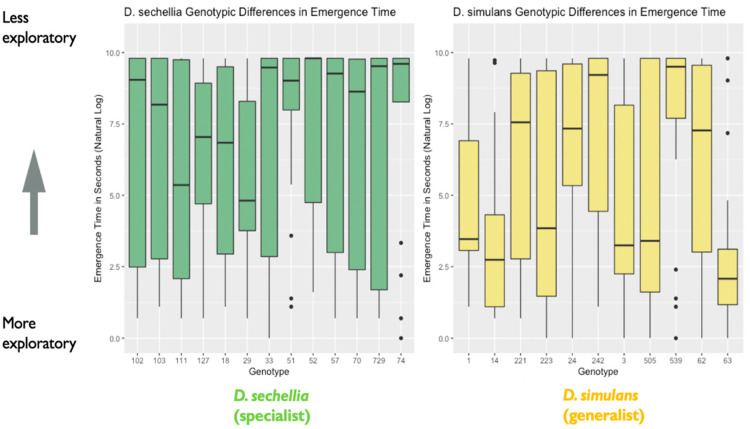
Genotypic differences in emergence time (in seconds): the plot represents genotypic differences in emergence time (in seconds), as noted in the main text. Data are from the first experiment measuring exploratory behavior. The y-axis represents the natural log of emergence time in seconds. A lower emergence time indicates higher exploratory behavior, while a higher emergence time represents lower exploratory behavior (*N* = 526 flies). The box in the plot represents the inter-quartile range of scores, including the middle 50% of scores for the group, with the median score represented by the middle quartile mark (or line). The whiskers represent the score ranges outside the inter-quartile range, including the top 25% of scores for the group in the upper whisker, and the bottom 25% of scores in the lower whisker. Our findings indicated significant genotypic differences in exploratory behavior.

We found support for sex differences in exploratory behavior, represented by the amount of time (in seconds) taken to emerge from a shelter into a novel environment. We found a significant effect of sex on emergence time (X^2^ = 4.06, degrees of freedom = 1, *p* = 0.04), with males showing significantly shorter emergence time than females (Figure 2). We found no effect of species on emergence time (X^2^ = 1.97, degrees of freedom = 1, *p* = 0.16), nor an effect of a two-way interaction between Species and Sex (X^2^ = 0.05, degrees of freedom = 1, *p* = 0.83). However, we did find evidence that genotypes differed significantly in emergence time based on the results of our likelihood ratio test, as including genotype as a random effect significantly improved model fit (likelihood ratio = 18.25, degrees of freedom = 1, *p* < 0.001). Additional follow-up models investigating the effect of genotype separately for each species indicated that the *D. simulans* (generalist) genotypes differed significantly in emergence time. A likelihood ratio test indicated that including genotype as a random effect significantly improved model fit for *D. simulans* (X^2^ = 19.12, degrees of freedom = 1, *p* < 0.0001). However, this was not the case for the *D. sechellia* (specialist) genotypes (X^2^ = 0.0005, degrees of freedom = 1, *p* = 0.98) (Figure 3).

### 3.2. Decision Making: Habitat Choice

#### 3.2.1. Species and Genotypes Differ in Habitat Choice

The majority of flies sampled both substrates at least once: the majority of the mean preference scores (73%, *N* = 382) were greater than 0 and less than 1. A total of 144 (27%) of the mean scores were either 0 (*N* = 104) or 1 (*N* = 40), indicating that we observed the fly sampling only one of the food types.

**Figure 4:**
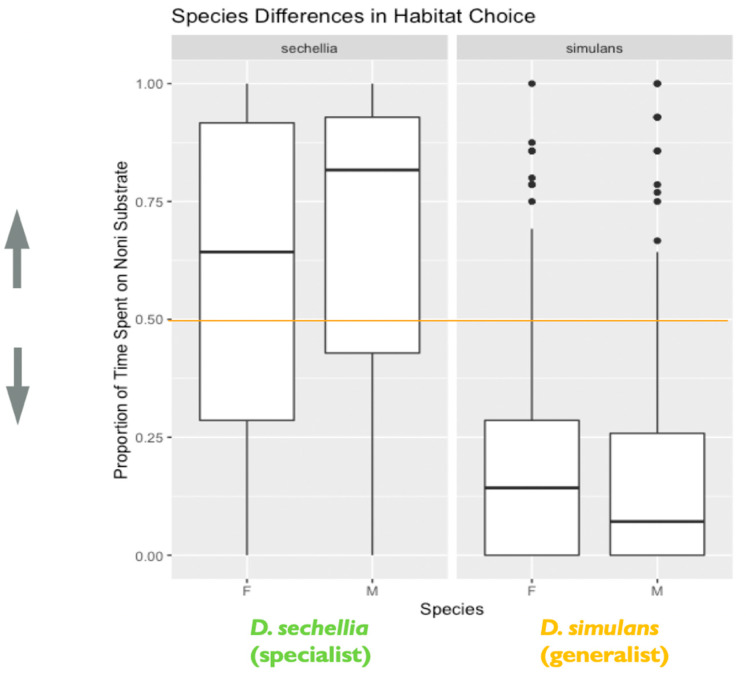
Species and sex differences in habitat choice: the plot represents species and sex differences in habitat (food substrate) choice, as noted in the main text. Data are from the second experiment measuring habitat choice as a representation of decision making. The y-axis represents the proportion of time spent on the imitation noni substrate. Lower values closer to 0 indicate a habitat preference for the plain substrate, while higher values closer to 1 indicate a habitat preference for the imitation noni substrate. A value on or near 0.5 (marked by the orange line) indicates no demonstrable habitat preference for either the plain or imitation noni substrates (*N* = 526 flies). The box in the plot represents the inter-quartile range of scores, including the middle 50% of scores for the group, with the median score represented by the middle quartile mark (or line). The whiskers represent the score ranges outside the inter-quartile range, including the top 25% of scores for the group in the upper whisker, and the bottom 25% of scores in the lower whisker. The dots beyond the whiskers represent outlier values. Our findings indicated that species differed significantly in habitat choice. Additionally, sex significantly influenced habitat choice, with males showing stronger habitat choice preference than females.

**Figure 5:**
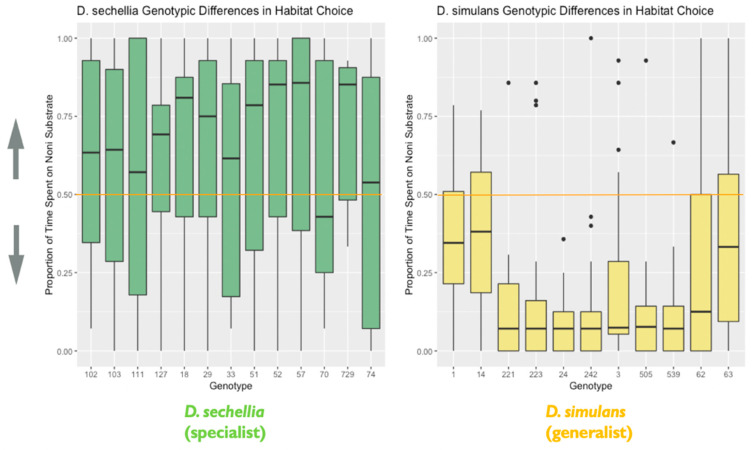
Genotypic differences in habitat choice: the plot represents genotypic differences in habitat (food substrate) choice, as noted in the main text. Data are from the second experiment measuring habitat choice. The y-axis represents the proportion of time spent on the imitation noni substrate. Lower values closer to 0 indicate a habitat preference for the plain substrate, while higher values closer to 1 indicate a habitat preference for the imitation noni substrate. A value on or near 0.5 (marked by the orange line) indicates no demonstrable habitat preference for either the plain or imitation noni substrates (*N* = 526 flies). The box in the plot represents the inter-quartile range of scores, including the middle 50% of scores for the group, with the median score represented by the middle quartile mark (or line). The whiskers represent the score ranges outside the inter-quartile range, including the top 25% of scores for the group in the upper whisker, and the bottom 25% of scores in the lower whisker. The dots beyond the whiskers represent outlier values. Our findings indicated that genotypes differed significantly in habitat choice.

We found support for species differences in habitat choice between *D. sechellia* and *D. simulans*. We observed a significant effect of species on habitat choice (X^2^ = 54.55, degrees of freedom = 1, *p* < 0.0001), with each species showing the hypothesized preference for their expected host (*D. simulans*/plain and *D. sechellia*/noni). *D. simulans*, the habitat generalist, showed stronger habitat choice preference for the plain fruit substrate than *D. sechellia*, the habitat specialist, showed for the imitation noni fruit substrate (Figure 4). Additionally, we found evidence that genotypes differed significantly in habitat choice, based on the results of the likelihood ratio test, as including genotype as a random effect significantly improved the fit of the final model (likelihood ratio = 10.29, degrees of freedom = 1, *p* = 0.001). Additional follow-up models investigating the effect of genotype separately for each species indicated that *D. simulans* genotypes differed significantly in habitat choice. A likelihood ratio test indicated that including genotype as a random effect significantly improved model fit for *D. simulans* (X^2^ = 34.26, degrees of freedom = 1, *p* < 0.0001). However, this was not the case for *D. sechellia* genotypes (X^2^ = 0, degrees of freedom = 1, *p* = 1) (Figure 5). 

#### 3.2.2. Sex Influences Habitat Choice, with Males Showing Stronger Habitat Choice than Females

**Figure 6:**
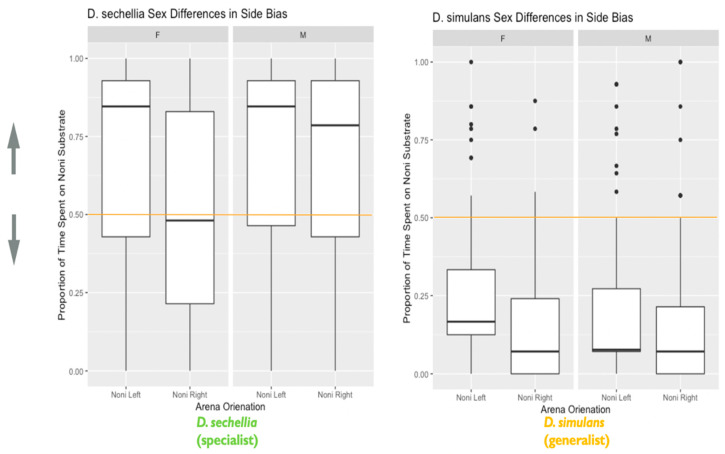
Species and sex differences in the effect of side bias on habitat choice: the plot represents species and sex differences in the effect of side bias on habitat choice, as described in the main text. Data are from the second experiment measuring habitat choice as a representation of decision making. The y-axis represents the proportion of time spent on the imitation noni substrate. Lower values closer to 0 indicate a habitat preference for the plain substrate, while higher values closer to 1 indicate a habitat preference for the imitation noni substrate. A value on or near 0.5 (marked by the orange line) indicates no demonstrable habitat preference for either the plain or imitation noni substrates. The x-axis is divided both by sex, as well as the arena orientation i.e., whether imitation noni substrate was on the left or right (*N* = 526 flies). The box in the plot represents the inter-quartile range of scores, including the middle 50% of scores for the group, with the median score represented by the middle quartile mark (or line). The whiskers represent the score ranges outside the inter-quartile range, including the top 25% of scores for the group in the upper whisker, and the bottom 25% of scores in the lower whisker. The dots beyond the whiskers represent outlier values. Our findings indicated that species and sexes differed significantly in habitat choice. Additionally, a left-side bias significantly influenced habitat choice, with females showing a stronger effect of left-side bias on habitat choice than males.

We observed a significant effect of sex on habitat choice (X^2^ = 10.13, degrees of freedom = 1, *p* = 0.002), as well as a significant two-way interaction between species and sex on habitat choice (X^2^ = 7.77, degrees of freedom = 1, *p* = 0.005). These results indicate that the males show a significantly stronger preference for their expected habitats, relative to their female counterparts (Figure 6), with *D. sechellia* (the specialist) showing a larger sex difference in habitat choice preference than *D. simulans* (the generalist).

#### 3.2.3. A Left-Side Bias Influences Habitat Choice, Particularly for Females

We also found evidence that the orientation of the food options within the habitat choice arena (whether imitation noni was on the left or right-hand side) significantly influenced habitat choice (X^2^ = 20.97, degrees of freedom = 1, *p* < 0.001). We observed evidence of a significant left-side bias in both species and sexes, which impacted habitat choice. This bias towards the left either facilitated or conflicted with habitat choice, depending on arena orientation. When the preferred habitat substrate was on the left, habitat choice was more accurate than when the preferred habitat substrate was on the right (Figure 6). These results indicate that a spatial bias can interfere with the outcome of this seemingly simple decision-making process. We also observed a significant two-way interaction between sex and habitat choice arena orientation on habitat choice (X^2^ = 4.52, degrees of freedom = 1, *p* = 0.03), with females showing a significantly stronger left-side bias than males (Figure 6). 

## 4. Discussion and Conclusions

Understanding how environmental factors influence decision making to produce variation in outcomes is important for understanding how decision making evolves, and for informing a more complete understanding of behavioral variation. Currently, there is a lack of understanding in what determines whether the fitness benefits of maximizing decision-making accuracy will outweigh the associated costs, and what is generating the variation in decision making observed in nature. In this study, we investigated whether differences in the degree of environmental predictability and variability experienced in nature were associated with differences in decision-making outcomes. In our species comparison of *D. sechellia* and *D. simulans*, we found that higher environmental unpredictability (in the form of higher habitat variability and wider diet breadth) was associated with higher habitat choice accuracy, but not with higher exploratory behavior. *D. simulans*, the generalist, showed higher habitat choice accuracy relative to *D. sechellia*, the specialist. While differences in environmental predictability were not associated with the predicted differences in exploratory behavior between these species, we did find that sex had a significant effect on exploratory behavior, with the males of both species being more exploratory than females. These findings are congruent with our hypothesis that higher environmental unpredictability should favor higher decision-making accuracy, but not our hypothesis that high environmental unpredictability should be associated with increased exploratory behavior and environmental sampling. Additionally, we found evidence that there was a significant effect of genotype on both exploratory behavior and habitat choice for *D. simulans*, but not *D. sechellia*. These findings indicate that *D. simulans* genotypes showed significant variation in exploratory behavior and habitat choice, while *D. sechellia* genotypes did not.

Additionally, we found that females showed lower habitat choice accuracy, relative to their male counterparts, and this effect was most pronounced in *D. sechellia* (the specialist). This finding was unexpected, particularly because female *Drosophila* must select habitats both for foraging and laying eggs and are therefore expected to be under strong selection to choose correctly. Female *D. sechellia* in particular gain fertility benefits from their noni host [24].

We also observed a significant effect of left-side bias on habitat choice for both species and sexes, reinforcing the accurate habitat choice when the accurate substrate was on the left and conflicting with the accurate habitat choice when the accurate substrate was on the right. The effect of left-side bias on habitat choice was most pronounced in females, particularly *D. sechellia* (the specialist). The stronger effect of left-side bias observed in females appeared to be a contributing factor to the sex differences in habitat choice accuracy, as females showed a stronger influence of side bias on their habitat choice, exhibiting significantly lower habitat choice accuracy when the preferred substrate was on the right.

While the predicted association between high habitat choice accuracy and high exploratory behavior was not observed between the two species, an association was observed for the males of each species, which showed both higher habitat choice accuracy and higher exploratory behavior, relative to their female counterparts. These sex differences were unexpected; but given that virgin females were used for this experiment, it is possible that the females were less motivated to both locate and choose the correct habitat. Additionally, male *Drosophila* are known to defend territories for mating purposes [48,49], perhaps leading to a higher motivation to both locate and select the ideal habitat. Future experiments measuring both virgin and mated flies could help elucidate whether differences in motivation (based on mated status) are a contributing factor to the observed differences in exploratory behavior and habitat choice accuracy between males and females.

Additionally, these findings indicate that certain cognitive biases, such as side bias, can be significantly influential in the decision-making outcomes of even seemingly simple decisions. This is surprising, given the simplicity of the choice (a two-choice assay in a small arena) and the ecological relevance of the habitat stimuli (particularly that the noni is toxic to *D. simulans*). Side bias, or “handedness”, has been observed in many other species [6,50,51,52,53,54,55,56], and previous work has indicated that it can influence the outcomes of various cognitive processes, such as foraging decisions and preference [6], spatial processing [57], and learning [50,58,59,60]. One proposed explanation for the existence of side bias is brain lateralization, which is the specialization of one hemisphere for a particular function, leaving the other hemisphere free to perform other or additional functions [61] Thus, the presence of a side bias could be indicative of a “good enough” approach to decision making, possibly providing a means for lowering the costs of decision making or managing errors under evolutionary constraints [20]. However, it is important to note that the observed side bias is likely not due to a true side, or space-use, bias, but a response to subtle differences in the environment that are not readily apparent to human observers, such as light or magnetic field. This is particularly because “side” is relative to an individual’s position or orientation within an environment, and subject to change as the individual moves. However, whether due to a true side bias or subtle environmental factors, the result remains surprising: when faced with a choice between two habitats, imperceptible (to humans) variation in seemingly unimportant environmental factors can dramatically influence choice. For example, *D. simulans* flies were apparently willing to spend time on food that is toxic to them (imitation noni), so long as that food was on the left. The mechanisms and evolution of this unexpected bias require further investigation.

This study, while powerful, was limited in a number of ways. First, the comparison was limited to only two species—one generalist and one specialist species. *D. sechellia* and *D. simulans* provide a strong model for investigating how recent divergence in habitat breadth influences the evolution of decision making, given that they are recently diverged and crossable, but occupy substantially different habitats. However, a range of physiological adaptations have accompanied the specialization of *D. sechellia* on noni, such as resistance to octanoic acid and hexanoic acid toxicity, changes in oviposition preference and olfaction, reduced ovariole number and egg production, and changes in larval morphology [24,37]. Additionally, differences in the potential costs associated with making an “incorrect” decision may have contributed to variation in decision-making accuracy between these species. *D. sechellia* can live on either noni or plain food (under laboratory conditions), but noni is poison to *D. simulans*. Therefore, it is possible that the observed differences in decision making between these species are due to something other than the differences in habitat breadth. Future work incorporating additional specialist/generalist comparisons within the *D. melanogaster* subgroup would help further demonstrate whether our findings are unique to the *D. simulans*/*D. sechellia* pair or are indicative of a larger pattern. Second, our measurement of exploratory behavior was limited to one measurement of emergence time and may not be the appropriate proxy for environmental sampling. The exploratory behavior observation period was capped at 5 h, with many individuals taking longer than 5 h to emerge. This possibly indicates a need for a longer experimental observation period, or a different measurement of exploratory behavior/environmental sampling. Additional information on exploratory behavior and environmental sampling could be obtained in future experiments by recording flies in the habitat choice arenas and measuring the amount of movement in addition to the amount of time spent on each of the habitat substrates. Third, while the plain fly medium recipe used for the assays differed from the plain fly medium these species were raised on, the plain fly food used for the trials was more similar to the rearing environment than the imitation noni substrate. Therefore, it is possible that the observed differences in habitat choice accuracy between these species are due, in part, to a familiarity effect. It is also possible that *D. sechellia* (the specialist) had decreased energy or motivation from not being raised on their preferred noni host. Future experiments raising flies on both plain and imitation noni substrates would help elucidate how rearing environment and prior experience influences habitat choice in these species.

In regard to our species comparison of *D. sechellia* and *D. simulans*, these findings support our hypothesis that higher environmental unpredictability should be associated with higher decision-making accuracy, but not our hypothesis that high environmental unpredictability should be associated with increased exploratory behavior and environmental sampling. Additionally, these findings demonstrate that cognitive biases, such as a side bias, can significantly influence the outcomes of even seemingly simple decisions. This study serves as an early step in investigating the environmental factors influencing the evolution of decision making, as well as informing a fuller understanding of why we observe behavioral variation among animals.

## References

[1] Arganda S., Perez-Escudero A., De Polavieja G.G. (2012). A common rule for decision making in animal collectives across species. Proc. Natl. Acad. Sci. USA.

[2] Blumstein D., Bouskila A. (1996). Assessment and Decision Making in Animals: A Mechanistic Model underlying Behavioral Flexibility Can Prevent Ambiguity. Oikos.

[3] Chittka L., Skorpupski P., Raine N.E. (2009). Speed-accuracy tradeoffs in animal decision making. Trends Ecol. Evol..

[4] Shettleworth S.J. (2001). Animal cognition and animal behaviour. Anim. Behav..

[5] Burns J.G., Rodd F.H. (2008). Hastiness, brain size and predation regime affect the performance of wild guppies in a spatial memory task. Anim. Behav..

[6] Jackson S., Nicolson S.W., Lotz C.W. (1998). Sugar Preferences and “Side Bias” in Cape Sugarbirds and Lesser Double-Collared Sunbirds. Auk.

[7] Moiron M., Mathot K.J., Dingemanse N.J. (2016). A multi-level approach to quantify speed-accuracy trade-offs in great tits (*Parus Major*). Behav. Ecol..

[8] Riebel K., Naguib M., Gil D. (2009). Experimental manipulation of the rearing environment influences adult female zebra finch song preferences. Anim. Behav..

[9] Rivalan M., Valton V., Series P., Marchand A.R., Dellu-Hagedorn F. (2013). Elucidating Poor Decision-Making in a Rat Gambling Task. PLoS ONE.

[10] Wood C.W., Wice E.W., Del Sol J., Sanderson B.J., Brodie E.D. (2018). Constraints Imposed by a Natural Landscape Override Offspring Fitness Effects to Shape Oviposition Decisions in Wild Forked Fungus Beetles. Am. Nat..

[11] Zentall T.R., Stagner J. (2010). Maladaptive choice behaviour by pigeons: An animal analogue and possible mechanism for gambling (sub-optimal human decision-making behaviour). Proc. R. Soc. B.

[12] Buchanan K.L., Grindstaff J.L., Pravosudov V.V. (2013). Condition dependence, developmental plasticity, and cognition: Implications for ecology and evolution. Trends Ecol. Evol..

[13] Dewitt T.J., Sih A., Wilson D.S. (1998). Costs and limits of phenotypic plasticity. Trends Ecol. Evol..

[14] Ricklefs R. (2004). The Cognitive Face of Avian Life Histories: The 2003 Margaret Morse Nice Lecture. Wilson J. Ornithol..

[15] Walker R., Burger O., Wagner J., Von Rueden C.R. (2006). Evolution of brain size and juvenile periods in primates. J. Hum. Evol..

[16] Caine N., Mundy N. (2000). Demonstration of a Foraging Advantage for Trichromatic Marmosets (*Callithrix geoffroyi*) Dependent on Food Colour. Proc. Biol. Sci..

[17] Caine N.G., Osorio D., Mundy N.I. (2010). A foraging advantage for dichromatic marmosets (*Callithrix geoffroyi*) at low light intensity. Biol. Lett..

[18] Snell-Rood E.C. (2013). An overview of the evolutionary causes and consequences of behavioral plasticity. Anim. Behav..

[19] Reed T.E., Waples R.S., Schindler D.E., Hard J.J., Kinnison M.T. (2010). Phenotypic plasticity and population viability: The importance of environmental predictability. Proc. R. Soc. B.

[20] Johnson D.D.P., Blumstein D.P., Fowler J.H., Haselton M.G. (2013). The evolution of error: Error management, cognitive constraints, and adaptive decision-making biases. Trends Ecol. Evol..

[21] Garrigan D., Kingan S.B., Geneva A.J., Andolfatto P., Clark A.G., Thornton K.R., Presgraves D.C. (2012). Genome sequencing reveals complex speciation in the *Drosophila simulans* clade. Genome Res..

[22] Kliman R.M., Andolfatto P., Coyne J.A., Depaulis F., Kreitman M., Berry A.J., McCarter J., Wakeley J., Hey J. (2000). The population genetics of the origin and divergence of the *Drosophila simulans* complex species. Genetics.

[23] Schrider D.R., Ayroles J., Matute D.R., Kern A.D. (2018). Supervised machine learning reveals introgressed loci in the genomes of *Drosophila simulans* and *D. sechellia*. PLoS Genet.

[24] Jones C.D. (2005). The genetics of adaptation in *Drosophila sechellia*. Genetica.

[25] Lachaise D., David J.R., Lemeunier F., Tsacas L. (1986). The reproductive relationships of *Drosophila sechellia* with *D. mauritiana*, *D. simulans*, and *D. melanogaster* from the Afrotropical region. Evolution.

[26] R’Kha S., Capy P., David J.R. (1990). Host-plant specialization in the *Drosophila melanogaster* species complex: A physiological, behavioral, and genetical analysis. Proc. Natl. Acad. Sci. USA.

[27] Powell J. (1997). Progress and Prospects in Evolutionary Biology: The Drosophila Model.

[28] Matute D.R., Gavin-Smyth J., Liu G. (2014). Variable post-zygotic isolation in *Drosophila melanogaster/D. simulans* hybrids. J. Evol. Biol..

[29] Davis J.M. (2007). Preference or desperation? Distinguishing between the natal habitat’s effects on habitat choice. Anim. Behav..

[30] Beckmann C., Biro P.A. (2013). On the Validity of a Single (Boldness) Assay in Personality Research. Ethology.

[31] Guillette L.M., Reddon A.R., Hurd P.L., Sturdy C.B. (2009). Exploration of a novel space is associated with individual differences in learning speed in black-capped chickadees, *Poecile atricapillus*. Behav. Process..

[32] Guillette L.M., Hahn A.H., Hoeschele M., Przyslupski A., Sturdy C.B. (2015). Individual differences in learning speed, performance accuracy and exploratory behavior in black-capped chickadees. Anim. Cogn..

[33] Perals D., Griffin A.S., Bartomeus I., Sol D. (2016). Revisiting the open-field test: What does it really tell us about animal personality?. Anim. Behav..

[34] Dworkin I., Jones C.D. (2009). Genetic Changes Accompanying the Evolution of Host Specialization in *Drosophila sechellia*. Genetics.

[35] Dekker T., Ibba I., Siju K.P., Stensmyr M.C., Hansson B.S. (2006). Olfactory Shifts Parallel Superspecialism for Toxic Fruit in *Drosophila melanogaster* Sibling, *D. sechellia*. Curr. Biol..

[36] Ibba I., Angioy A.M., Hansson B.S., Dekker T. (2010). Macroglomeruli for fruit odors change blend preference in *Drosophila*. Sci. Nat..

[37] Lavista-Llanos S., Svatos A., Kai M., Riemensperger T., Birman S., Stensmyr M.C., Hansson B.S. (2014). Dopamine drives *Drosophila sechellia* adaptation to its toxic host. eLife.

[38] Prieto-Godino L.L., Rytz R., Cruchet S., Bargeton B., Abuin L., Silbering A.F., Ruta V., Peraro M.D., Benton R. (2017). Evolution of Acid-Sensing Olfactory Circuits in Drosopholids. Neuron.

[39] Auer T.O., Khallaf M.A., Silbering A.F., Zappia G., Ellis K., Alvarez-Ocana R., Arguello J.R., Hansson B.S., Jefferis G.S.X.E., Caron S.J.C. (2020). Olfactory receptor and circuit evolution promote host specialization. Nature.

[40] Egan L.C., Bloom P., Santos L.R. (2010). Choice-induced preferences in the absence of choice: Evidence from a blind two choice paradigm with young children and capuchin monkeys. J. Exp. Soc. Psychol..

[41] Jozefowiez J., Staddon J.E.R., Cerutti D.T. (2009). Metacognition in animals: How do we know that they know?. Comp. Cogn. Behav. Rev..

[42] Roche J.P., Timberlake W., McCloud C. (2013). Sensitivity to variability in food amount: Risk aversion is seen in discrete-choice, but not in free-choice, trials. J. Chem. Inf. Model..

[43] Catania A.C., Sagvolden T. (1980). Preference for free choice over forced choice in pigeons. J. Exp. Anal. Behav..

[44] Therneau T. coxme: Mixed Effects Cox Models 2018. https://cran.r-project.org/web/packages/coxme/vignettes/coxme.pdf.

[45] Pankratz V.S., De Andrade M., Therneau T.M. (2005). Random-effects Cox proportional hazards model: General variance components methods for time-to-event data. Genet. Epidemiol..

[46] Fox J., Weisberg S. (2010). An R Companion to Applied Regression.

[47] Bolker B.M., Brooks M.E., Clark C.J., Geange S.W., Poulsen J.R., Stevens M.H.H., White J.S. (2008). Generalized linear mixed models: A practical guide for ecology and evolution. Trends Ecol. Evol..

[48] Saltz J.B., Foley B.R. (2011). Natural Genetic Variation in Social Niche Construction: Social Effects of Aggression Drive Disruptive Sexual Selection in *Drosophila Melanogaster*. Am. Nat..

[49] Saltz J.B. (2013). Genetic composition of social groups influences male aggressive behaviour and fitness in natural genotypes of *Drosophila melanogaster*. Proc. R. Soc. B.

[50] Alves C., Chichery R., Boal J.G., Dickel L. (2007). Orientation in the cuttlefish *Sepia officinalis*: Response versus place learning. Anim. Cogn..

[51] Kight S.L., Steelman L., Coffey G., Lucente J., Castillo M. (2008). Evidence of population-level lateralized behaviour in giant water bugs, Belostoma flumineum Say (Heteroptera: Belostomatidae): T-maze turning is left biased. Behav. Process..

[52] Collins R.L. (1975). When left-handed mice live in right-handed worlds. Science.

[53] Andrade C., Alwarshetty M., Sudha S., Suresh Chandra J. (2001). Effect of innate direction bias on T-maze learning in rats: Implications for research. J. Neurosci. Methods.

[54] Castellano M.A., Diaz-Palarea M.D., Rodriguez M., Barroso J. (1987). Lateralization in male rats and dopaminergic system: Evidence of right-side population bias. Physiol. Behav..

[55] Glick S.D., Ross D.A. (1981). Lateralization of function in the rat brain. Trends Neurosci..

[56] Sherman G.F., Garbanati J.A., Rosen G.D., Yutzey D.A., Denenberg V.H. (1980). Brain and Behavioral Asymmetries for Spatial Preference in Rats. Brain Res..

[57] Doria M.D., Morand-Ferron J., Bertram S.M. (2019). Spatial cognitive performance is linked to thigmotaxis in field crickets. Anim. Behav..

[58] Anfora G., Frasnelli E., Maccagnani B., Rogers L.J., Vallortigara G. (2009). Behavioural and electrophysiological lateralization in a social (*Apis mellifera*) but not in a non-social (*Osmia cornuta*) species of bee. Behav. Brain Res..

[59] Letzkus P., Ribi W.A., Wood J.T., Zhu H., Zhang S.W., Srinivasan M.V. (2006). Lateralization of Olfaction in the Honeybee *Apis mellifera*. Curr. Biol..

[60] Vallortigara G., Andrew R.J. (1994). Olfactory lateralization in the chick. Neuropsychologia.

[61] Vallortigara G., Rogers L.J. (2005). Survival with an asymmetrical brain: Advantages and disadvantages of cerebral lateralization. Behav. Brain Sci..

